# Notch Inhibition Promotes Differentiation of Liver Progenitor Cells into Hepatocytes via *sox9b* Repression in Zebrafish

**DOI:** 10.1155/2019/8451282

**Published:** 2019-03-12

**Authors:** Jacquelyn O. Russell, Sungjin Ko, Satdarshan P. Monga, Donghun Shin

**Affiliations:** ^1^Department of Pathology, University of Pittsburgh, Pittsburgh, USA; ^2^Department of Developmental Biology, University of Pittsburgh, Pittsburgh, USA; ^3^Pittsburgh Liver Research Center, University of Pittsburgh, Pittsburgh, USA; ^4^Department of Medicine, University of Pittsburgh, Pittsburgh, USA; ^5^McGowan Institute for Regenerative Medicine, University of Pittsburgh, Pittsburgh, USA

## Abstract

Liver regeneration after most forms of injury is mediated through the proliferation of hepatocytes. However, when hepatocyte proliferation is impaired, such as during chronic liver disease, liver progenitor cells (LPCs) arising from the biliary epithelial cell (BEC) compartment can give rise to hepatocytes to mediate hepatic repair. Promotion of LPC-to-hepatocyte differentiation in patients with chronic liver disease could serve as a potentially new therapeutic option, but first requires the identification of the molecular mechanisms driving this process. Notch signaling has been identified as an important signaling pathway promoting the BEC fate during development and has also been implicated in regulating LPC differentiation during regeneration. SRY-related HMG box transcription factor 9 (Sox9) is a direct target of Notch signaling in the liver, and Sox9 has also been shown to promote the BEC fate during development. We have recently shown in a zebrafish model of LPC-driven liver regeneration that inhibition of Hdac1 activity through MS-275 treatment enhances *sox9b* expression in LPCs and impairs LPC-to-hepatocyte differentiation. Therefore, we hypothesized that inhibition of Notch signaling would promote LPC-to-hepatocyte differentiation by repressing *sox9b* expression in zebrafish. We ablated the hepatocytes of *Tg(fabp10a:CFP-NTR)* larvae and blocked Notch activation during liver regeneration through treatment with *γ*-secretase inhibitor LY411575 and demonstrated enhanced induction of Hnf4a in LPCs. Alternatively, enhancing Notch signaling via Notch3 intracellular domain (N3ICD) overexpression impaired Hnf4a induction. Hepatocyte ablation in *sox9b* heterozygous mutant embryos enhanced Hnf4a induction, while BEC-specific Sox9b overexpression impaired LPC-to-hepatocyte differentiation. Our results establish the Notch-Sox9b signaling axis as inhibitory to LPC-to-hepatocyte differentiation in a well-established *in vivo* LPC-driven liver regeneration model.

## 1. Introduction

The liver is the only human internal organ capable of regeneration, and after most forms of acute injury, this regeneration is mediated by the proliferation of the differentiated epithelial cells of the liver, namely, hepatocytes or biliary epithelial cells (BECs). However, during prolonged chronic liver injury, this innate regenerative capacity may be exhausted, leading to progression to end-stage liver disease, cirrhosis, and liver failure [1]. A common feature of chronic liver disease in human patients is the ductular reaction or proliferation of “reactive” BECs, with the degree of BEC expansion correlating with the severity of liver injury [2, 3]. Within the ductular reaction, there are thought to be liver progenitor cells (LPCs), bipotent cells capable of differentiating into hepatocytes or BECs. Although LPCs remain a controversial topic, there is evidence of BEC-to-hepatocyte differentiation in animal models with near-total ablation of hepatocytes [4–6], impaired hepatocyte proliferation during severe liver injury [7–10], and prolonged chronic liver injury [11] and in human patients with cirrhosis [12] or massive hepatic necrosis [13]. With a dearth of treatments for end-stage liver disease besides liver transplantation, the idea of promoting LPC-to-hepatocyte differentiation as a new source of hepatic parenchyma is an attractive option. However, the molecular mechanisms underpinning LPC-to-hepatocyte differentiation remain largely unknown, precluding the development of such therapies.

The Notch signaling pathway is an important pathway during liver development, where its activation in fetal hepatoblasts promotes biliary differentiation and bile duct morphogenesis [14–16]. There are four Notch receptors (Notch1-4) and two families of ligands (Jagged-1 and -2 and Delta-like-ligand 1, 3, and 4) in mammals. Notch signaling is a form of juxtacrine signaling where the Notch receptor located in the cell membrane of one cell binds its ligand expressed on the membrane of an adjacent cell, activating the Notch receptor and leading to proteolytic cleavage of the Notch intracellular domain (NICD) by *γ*-secretase. The NICD translocates to the nucleus, where it interacts with the DNA-binding protein recombination signal-binding protein immunoglobulin kappa J (Rbpj) to mediate target gene transcription [17]. In addition to its essential role in liver development, Notch signaling has been implicated in liver regeneration, where several groups have implicated a role for activation of Notch signaling in promoting LPC differentiation to BECs while inhibiting hepatocyte differentiation *in vitro* [16, 18, 19]. Similarly, inhibition of Notch signaling blocked BEC differentiation and promoted LPC-to-hepatocyte differentiation *in vitro* [18–20], while *in vivo* inhibition of Notch signaling impaired BEC proliferation after bile duct ligation [21] and inhibited LPC differentiation in the rat 2-acetylaminofluorine combined with partial hepatectomy model [22]. Work in the zebrafish hepatocyte ablation model showed that dedifferentiation of BECs into LPCs required Notch-dependent Sox9b activation [5], while high levels of Notch signaling in LPCs prevented LPC-to-hepatocyte differentiation [6]. On the other hand, deletion of Rbpj from BECs and subsequent exposure to the 3,5-diethoxycarbonyl-1,4-dihydrocollidine (DDC) diet, which induces cholangitis, impaired the ductular reaction and reduced the expression of mature biliary markers but was not sufficient to induce BEC-to-hepatocyte conversion [23]. Together, these data implicate a role for Notch signaling in the regulation of BECs and LPCs during liver regeneration, although the downstream effectors critical to this process remain to be clearly elucidated.

During liver development, SRY-related HMG box transcription factor 9 (Sox9) is considered to be the earliest marker of commitment to the biliary fate. Liver-specific deletion of *Sox9* in mice results in delayed biliary development [24], while zebrafish homozygous *sox9b* mutants exhibit improper differentiation of progenitors into the hepatic or pancreatic fates and also display cholestasis from impaired intrahepatic duct formation during early liver development [25]. Sox9 is expressed by BECs at baseline in adult mice and in reactive BECs during liver injury, but there is ectopic expression of Sox9 in hepatocytes during forms of cholestatic liver injury such as biliary atresia [26] and ornithine transcarbamylase deficiency [27] in humans and DDC diet [28, 29] and rarely after choline-deficient and ethionine-supplemented diet [30] in rodents. When Sox9+ hepatocytes were isolated from injured mouse livers, they formed organoids capable of differentiating into both BECs and hepatocytes [28, 30], although clonal lineage tracing suggested Sox9+ LPCs rarely produce hepatocytes *in vivo* [30]. In line with this observation, expression of Sox9 was shown to be downregulated in LPCs differentiating towards the hepatocyte lineage [27], and CD24+ bipotential Sox9+ hepatocytes displayed downregulation of hepatocyte markers [31]. These data suggest that Sox9 is an important regulator of hepatocyte/biliary phenotype conversion during both liver development and regeneration.

Sox9 has also been shown to be a direct target of Notch signaling in the liver. When NICD was overexpressed during mouse liver development, there was dramatic upregulation in *Sox9* expression in the livers of newborn pups [15]. Additionally, Notch1 was shown to directly bind to Rbpj-binding sites identified in the *Sox9* promoter [15]. In *sox9b* mutant zebrafish embryos, it was shown that Sox9b was required for the maintenance, but not the initiation, of Notch signaling in intrahepatic biliary cells during liver development [25]. Despite the known importance of Sox9 in regulating cell fate in hepatoblasts during development, little is known about the role of Sox9 in LPC cell fate regulation during regeneration. We have previously established an *in vivo* zebrafish model of extreme hepatocyte ablation such that liver regeneration is mediated by LPC-to-hepatocyte differentiation [4]. This model system is amenable to drug screening to identify the mediators of LPC proliferation and differentiation; we have previously shown that bromodomain and extraterminal (BET) protein inhibition blocked BEC dedifferentiation into LPCs [32] and selective bone morphogenetic protein (BMP) inhibitor DMH1 impaired LPC-to-hepatocyte differentiation [33], and we recently demonstrated that inhibition of histone deacetylase 1 (Hdac1) activity impaired LPC-to-hepatocyte differentiation through the enhancement of *sox9b* expression [34]. Given that Sox9 is a direct target of Notch signaling in the liver, and the plethora of *in vitro* data suggest a role for Notch signaling in LPC differentiation, we hypothesized that inhibition of Notch signaling would promote LPC-to-hepatocyte differentiation through the repression of *Sox9* expression. Here, we utilized LY411575, a small molecule inhibitor of *γ*-secretase which blocks Notch activation, in our zebrafish hepatocyte ablation model to show that inhibition of Notch signaling promotes Hnf4a induction in LPCs, while overexpression of NICD impairs Hnf4a induction. We further show that deletion of one allele of *sox9b* in zebrafish augments Hnf4a induction in LPCs, while BEC-specific overexpression of Sox9b impairs LPC-to-hepatocyte differentiation. Finally, we show that inhibition of Notch signaling is sufficient to rescue the impaired LPC-to-hepatocyte differentiation in larvae with inhibition of Hdac1 activity, but does not rescue the defects in BET inhibitor or DMH1-treated larvae. Our work clearly establishes the importance of the Notch-Sox9 signaling axis during *in vivo* LPC differentiation.

## 2. Results

### 2.1. LY411575 Treatment Promotes Hnf4a Induction in BECs during BEC-Mediated Regeneration

**Figure 1:**
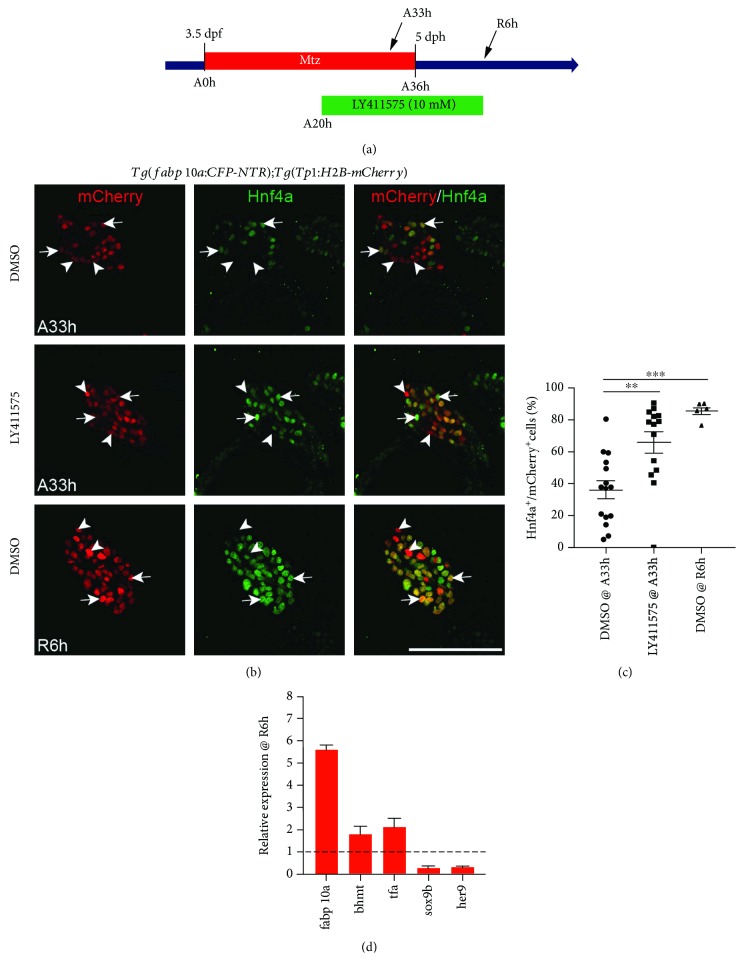
Pharmacological Notch inhibition promotes differentiation of LPCs into hepatocytes. (a) Experimental scheme illustrating the periods of MTZ and LY411575 treatment and analysis stages (arrow). (b) Single-optical section images showing Hnf4a and *Tp1:*H2B-mCherry expression in regenerating livers at A33h and R6h. Arrows point to Hnf4a^+^ BEC-derived cells; arrowheads point to Hnf4a^−^ BEC-derived cells. (c) Quantification of the percentage of Hnf4a^+^ cells among BEC-derived cells, as shown in (b). (d) qPCR data showing the relative expression levels of *fabp10a*, *bhmt*, *tfa*, and *sox9b* between DMSO- and LY411575-treated regenerating livers at R6h. Scale bar: 100 *μ*m; error bars: ±SEM. ^∗∗^, *P* < 0.01; ^∗∗∗^*P* < 0.001.

In order to assess the role of Notch signaling in LPC-driven liver regeneration, we utilized our previously published hepatocyte ablation model [4]. We used the *Tg(fabp10a:CFP-NTR)* line, which expresses nitroreductase (NTR) under the hepatocyte-specific *fabp10a* promoter (ref). When these larvae are exposed to nontoxic metronidazole (MTZ) for 36 hours (A36h) from 3.5 to 5 days postfertilization (dpf), NTR expression in hepatocytes converts MTZ into a cytotoxic compound, leading to near-total hepatocyte ablation. We also used the *Tg(Tp1:H2B-mCherry)* line to mark BECs, as the mCherry expression is driven by the *Tp1* promoter containing Notch-responsive elements [35]. The H2B-mCherry protein's stability allows for tracing of BEC cell fate over several cell divisions [35]. After hepatocyte ablation, removal of MTZ allows liver regeneration to commence, during which BEC-derived LPCs differentiate into hepatocytes [4, 5]. To determine the effect of Notch inhibition on the LPC-to-hepatocyte differentiation process, we treated the larvae with *γ*-secretase inhibitor LY411575 for up to 22 hours, starting from 20 hours into MTZ ablation (A20h) until 6 hours after MTZ washout (R6h) (Figure 1(a)). After 13 hours of LY411575 treatment (A33h), there was a significant increase in the number of *Tp1*:H2B-mCherry^+^/Hnf4a^+^ cells in LY411575-treated livers compared to dimethyl sulfoxide- (DMSO-) treated controls (Figures 1(b) and 1(c)). Hnf4a is not expressed in BECs of nonablated livers [4]. In fact, the number of *Tp1*:H2B-mCherry^+^/Hnf4a^+^ cells in LY411575-treated regenerating larvae at A33h was not significantly different from that in DMSO-treated regenerating larvae at R6h (Figures 1(b) and 1(c); *P* = 0.167). The total number of *Tp1*:H2B-mCherry^+^ cells were comparable between DMSO- and LY411575-treated regenerating livers at A33h (Figure 1(b)), suggesting that the increase in *Tp1*:H2B-mCherry^+^/Hnf4a^+^ cells was not due to BEC proliferation. Assessment of gene expression at R6h revealed an increase in hepatocyte markers *fabp10a*, *bhmt*, and *tfa* and a decrease in *sox9b* and Notch target gene *her9* expression in LY411575-treated regenerating livers compared to DMSO controls (Figure 1(d)). Together, these results suggest that inhibition of Notch signaling promotes LPC-to-hepatocyte differentiation during BEC-mediated liver repopulation.

### 2.2. N3ICD Overexpression Inhibits Hnf4a Induction in BECs during BEC-Mediated Regeneration

**Figure 2:**
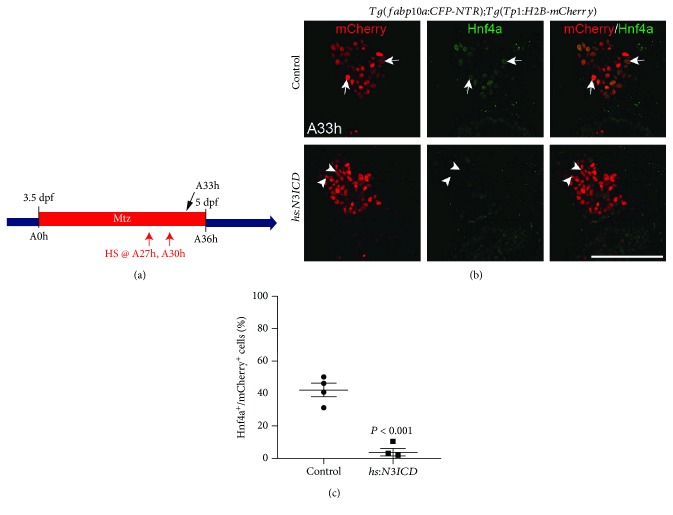
N3ICD overexpression impairs Hnf4a induction in BECs. (a) Experimental scheme illustrating the period of MTZ treatment and the stages of heat-shock (red arrows) and analysis (black arrow). (b) Single-optical section images showing Hnf4a and *Tp1:*H2B-mCherry expression in regenerating livers at A33h. Arrows point to Hnf4a^+^ BEC-derived cells with round nuclei; arrowheads point to Hnf4a^−^ BEC-derived cells with elongated nuclei. (c) Quantification of the percentage of Hnf4a^+^ cells among BEC-derived cells, as shown in B. Scale bar: 100 *μ*m; error bars: ±SEM.

We next assessed the effect of enhanced Notch signaling during LPC-driven regeneration using the *Tg(hs:N3ICD)* line, where the inducible expression of N3ICD is under the control of the heat shock protein 70l promoter [36]. Heat shock was applied at the A27h and A30h time points to induce N3ICD expression, and the larvae were analyzed at A33h (Figure 2(a)). In control larvae, Hnf4a expression was evident in approximately 40% of *Tp1*:H2B-mCherry^+^ cells, but the number of *Tp1*:H2B-mCherry^+^/Hnf4a^+^ cells was dramatically reduced in N3ICD-expressing larvae (Figures 2(b) and 2(c)). Again, the total number of *Tp1*:H2B-mCherry^+^ cells was comparable between the two groups. We also noted that as assessed by *Tp1*:H2B-mCherry expression patterns, more *Tp1*:H2B-mCherry^+^ cells in N3ICD-expressing larvae than in controls exhibited an elongated shape of their nuclei (Figure 2(b), arrowheads), resembling BEC nuclei, and most *Tp1*:H2B-mCherry^+^ cells in the controls exhibited a round shape of their nuclei (Figure 2(b), arrows), a feature reminiscent of hepatocyte nuclei. These N3ICD overexpression data suggest that enhancement of Notch signaling impairs LPC-to-hepatocyte differentiation in our zebrafish model.

### 2.3. Hnf4a Induction in BECs Is Enriched in *sox9b* Heterozygous Mutant Zebrafish

**Figure 3:**
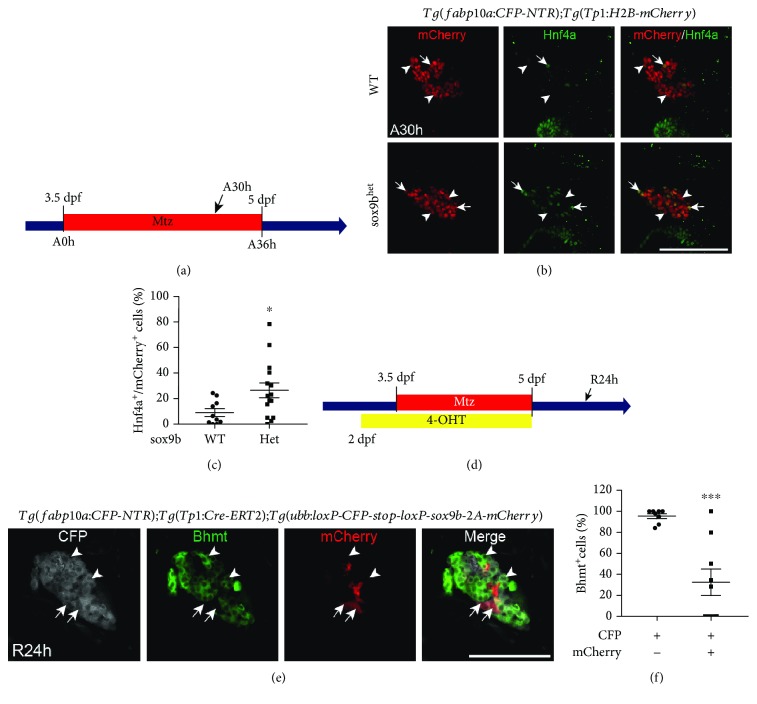
Sox9b suppresses LPC-to-hepatocyte differentiation. (a) Experimental scheme illustrating the period of MTZ treatment and analysis stage (arrow). (b) Single-optical section images showing Hnf4a and *Tp1:*H2B-mCherry expression in regenerating livers at A30h. Arrows point to Hnf4a^+^ BEC-derived cells; arrowheads point to Hnf4a^−^ BEC-derived cells. (c) Quantification of the percentage of Hnf4a^+^ cells among BEC-derived cells, as shown in (b). (d) Experimental scheme illustrating the periods of MTZ and 4-OHT treatment and analysis stage (arrow). (e) Single-optical section images showing *fabp10a:*CFP-NTR, Bhmt, and mCherry expression in regenerating livers at A24h. mCherry expression was revealed by anti-mCherry immunostaining. Arrows point to CFP^+^/Bhmt^−^/mCherry^+^ BEC-derived cells; arrowheads point to CFP^+^/mCherry^−^/Bhmt^+^ hepatocytes. (f) Quantification of the percentage of Bhmt^+^ hepatocytes between CFP^+^/mCherry^−^ and CFP^+^/mCherry^+^ populations, as shown in (e). Scale bars: 100 *μ*m; error bars: ±SEM. ^∗^*P* < 0.05; ^∗∗∗^*P* < 0.001.

As *sox9b* is a direct downstream target gene of Notch signaling [25], we next assessed if reduction of *sox9b* would be sufficient to enhance LPC-to-hepatocyte differentiation, as observed in LY411575-treated regenerating livers. To this end, we utilized *sox9b* heterozygous mutant zebrafish, as they undergo normal liver development in the absence of a challenge [25]. We decided to analyze these larvae at the A30h time point during MTZ hepatocyte ablation (Figure 3(a)), as this stage marks the start of Hnf4a induction in BECs. At this time point, there were significantly more *Tp1*:H2B-mCherry^+^/Hnf4a^+^ cells in *sox9b^+/-^* mutants than in their wild-type siblings (Figures 3(b) and 3(c)), indicating facilitated Hnf4a induction in BECs by Sox9b haploinsufficiency.

### 2.4. BEC-Specific Sox9b Overexpression Impairs LPC-to-Hepatocyte Differentiation

To determine if overexpression of Sox9b was sufficient to block LPC-to-hepatocyte differentiation, we utilized *Tg(fabp10a:CFP-NTR)*; *Tg(Tp1:CreERT2)*; *Tg(ubb:loxP-CFP-loxP-sox9b-2A-mCherry)* larvae, which upon administration of 4-hydroxytamoxifen (4-OHT) overexpresses Sox9b specifically in BECs [37]. To this end, we treated the larvae with 4-OHT from 2 to 5 dpf, overlapping with MTZ-induced hepatocyte ablation (Figure 3(d)). To assess LPC-to-hepatocyte differentiation, we examined the expression of mCherry (Sox9b-overexpressing cells), hepatocyte marker Bhmt, and CFP (derived from the *fabp10a:CFP-NTR* transgene) in livers at the R24h time point, at which LPC-derived hepatocytes are evident [4]. Quantification of the percentage of Bhmt^+^ cells between CFP^+^/mCherry^−^ and CFP^+^/mCherry^+^ populations revealed significantly fewer Bhmt^+^ cells in the CFP^+^/mCherry^+^ population (Figure 3(f)), suggesting that Sox9b overexpression inhibits LPC-to-hepatocyte differentiation. The mCherry^+^/Bhmt^+^ hepatocytes observed in this model may be potentially derived from cells in which Sox9b overexpression was induced after Hnf4a induction or in cells where there was not sufficient Sox9b expression to impair LPC-to-hepatocyte differentiation, as 4-OHT was administered until the end of the ablation period. Together with the data from the *sox9b* mutants, these results indicate that Sox9b expression in BECs is inhibitory to LPC-to-hepatocyte differentiation.

### 2.5. LY411575 Treatment Rescues Defective BEC-to-Hepatocyte Transition in MS-275-Treated, but Not JQ1- or DMH1-Treated, Regenerating Livers

**Figure 4:**
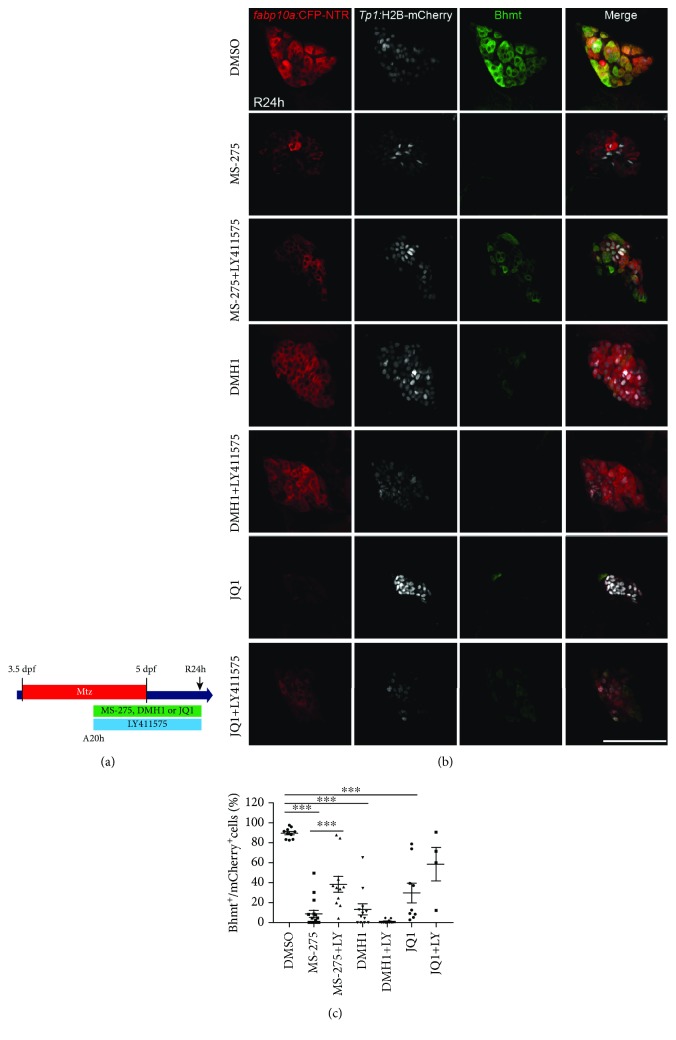
LY411575 treatment rescues a defect in LPC-to-hepatocyte differentiation in MS-275-treated regenerating larvae. (a) Experimental scheme illustrating the periods of MTZ and compound treatment and analysis stage (arrow). (b) Single-optical section images showing *fabp10a:*CFP-NTR, *Tp1:*H2B-mCherry, and Bhmt expression in regenerating livers at R24h. (c) Quantification of the percentage of Bhmt^+^ hepatocytes among BEC-derived cells, as shown in (b). Scale bar: 100 *μ*m; error bars: ±SEM. ^∗∗∗^*P* < 0.001.

To further prove the direct mechanistic link between Notch signaling and Sox9b in regulating LPC differentiation, we sought to determine if inhibition of Notch signaling could rescue the differentiation defect in larvae treated with Hdac1 inhibitor MS-275, which impairs LPC-to-hepatocyte differentiation through enhanced *sox9b* expression [34]. To this end, we treated *Tg(fabp10a:CFP-NTR)*;*Tg(Tp1:H2B-mCherry)* larvae with a various combination of compounds and assessed the extent of LPC-to-hepatocyte differentiation at the R24h time point (Figure 4(a)). We stained for Bhmt to identify LPC-derived hepatocytes, which were evident in DMSO-treated control larvae (Figure 4(b)). In larvae treated with MS-275 alone, there were significantly fewer Bhmt^+^/H2B-mCherry^+^ cells, indicating impaired LPC-to-hepatocyte differentiation. However, when larvae were cotreated with MS-275 and LY411575, at R24h, there was a significant increase in the number of Bhmt^+^/H2B-mCherry^+^ cells (Figure 4(c)), indicating a rescue of defective LPC-to-hepatocyte differentiation induced by MS-275 treatment. If the mechanism of Notch inhibition promoting LPC-to-hepatocyte differentiation is through the reduction of *sox9b* expression, then other compounds that potentially inhibit LPC differentiation through mechanisms other than enhancement of *sox9b* expression should not be affected by LY411575 treatment. Therefore, we utilized DMH1, a selective BMP inhibitor which inhibits LPC-to-hepatocyte differentiation [33]. Treatment with DMH1 alone impaired Bhmt expression, and cotreatment of DMH1 and LY411575 exhibited a similar impairment of Bhmt induction (Figures 4(b) and 4(c)), indicating that inhibition of Notch signaling does not rescue the LPC-to-hepatocyte differentiation defect induced by impaired BMP signaling. When larvae were treated with JQ1, a BET protein inhibitor, there was defective BEC dedifferentiation into LPCs, as evidenced by diminished CFP and Bhmt expression at R24h (Figure 4(b)). Cotreatment with LY411575 did not significantly increase the number of Bhmt^+^/H2B-mCherry^+^ cells in JQ1-treated larvae (Figure 4(c)), indicating that inhibition of Notch signaling is not sufficient to rescue the LPC defect in larvae with BET protein inhibition. Together, these data indicate that LY411575 is only capable of ameliorating defective LPC-to-hepatocyte differentiation if this defect is caused by the enhanced *sox9b* expression, as is the case for MS-275-treated regenerating larvae but not DMH1- or JQ1-treated larvae.

## 3. Discussion

In this study, we demonstrate that suppression of the Notch-Sox9b signaling axis promotes LPC-to-hepatocyte differentiation in our zebrafish hepatocyte ablation model. Given the robust dedifferentiation of BECs into LPCs after hepatocyte ablation and the highly reproducible and coordinated differentiation of LPCs into either hepatocytes or BECs [4], this model allows for careful assessment of the molecular mechanisms of LPC differentiation. Additionally, the large clutch sizes, rapid growth in water, and optical transparency of zebrafish embryos [38] make it very easy to use small molecule inhibitors to modulate specific signaling pathways during the key phases of liver regeneration [32–34]. To this end, we used the *γ*-secretase inhibitor LY411575 to demonstrate enhanced induction of the hepatocyte marker Hnf4a in BECs during early liver regeneration. We further showed that activation of Notch signaling through N3ICD overexpression blocked Hnf4a induction. We additionally demonstrated that *sox9b^+/-^* mutant larvae displayed facilitated Hnf4a induction, while BEC-specific Sox9b overexpression reduced the expression of a hepatocyte marker Bhmt, revealing that modulation of Sox9b mimics Notch modulation during LPC-driven liver regeneration. Finally, we mechanistically link Notch and Sox9b by showing that LY411575 cotreatment rescues the *sox9b* overexpression-mediated LPC-to-hepatocyte differentiation defect in MS-275-treatedregenerating larvae [34]. We provided supporting evidence that this rescue was Sox9b dependent, as LY411575 cotreatment did not rescue the LPC differentiation defects in DHM1- [33] or JQ1-treated larvae [32].

Notch signaling promotes bile duct morphogenesis during rodent liver development [14–16]. Notch signaling is also important in human liver development, as Alagille syndrome patients have mutations in Notch signaling components, such as *Jagged1* [39, 40] and *Notch2* [41], and the majority of patients display clinical manifestations of neonatal jaundice, cholestasis, and paucity of intrahepatic bile ducts [42]. Consistent with its role in biliary development, Notch signaling has been shown to promote BEC or LPC proliferation during liver injury [6, 21, 23]. Many studies have also demonstrated that *in vitro* inhibition of Notch signaling is required for LPC-to-hepatocyte differentiation [18–20], which is confirmed by the *in vivo* data presented in this current study. Expansion of reactive BECs is a common feature of chronic liver disease in humans [3], and the cells of the ductular reaction are known to secrete proinflammatory and profibrogenic cytokines [43]. As the extent of the ductular reaction correlates with the severity of liver injury [2], it has been theorized that promotion of LPC-to-hepatocyte differentiation would serve two purposes: to [1] reduce the profibrogenic ductular reaction and [2] provide a source of hepatocytes. To this end, inhibition of Notch signaling in patients with chronic liver injury could potentially reduce the number of reactive BECs, thereby reducing hepatic fibrogenesis, as well as promote LPC-to-hepatocyte differentiation. In support of this hypothesis, a zebrafish hepatic fibrosis model found that antagonism of Notch signaling promoted LPC-to-hepatocyte differentiation [6], and a mouse model of steatohepatitis found that macrophage expression of Jagged1 promoted differentiation of LPCs into BECs, while expression of NUMB that impairs Notch signaling promoted differentiation of LPCs into hepatocytes [44]. However, other groups have reported that Notch signaling was required for the dedifferentiation of BECs into LPCs in the zebrafish hepatocyte ablation model [5] and that inhibition of Notch signaling was not sufficient to promote LPC-to-hepatocyte differentiation during cholestatic liver injury [23]. Additionally, inhibition of Notch signaling may impair liver regeneration because Notch signaling has been reported to promote hepatocyte proliferation after partial hepatectomy in rats [45, 46], although a different group reported that overexpression of Rbpj in a hepatocyte cell line reduced hepatocyte proliferation [16]. This mixed evidence about the efficacy of Notch inhibition in promoting liver regeneration indicates that more careful *in vivo* analyses of Notch inhibition in clinically relevant models of chronic liver injury are necessary before Notch inhibitors can be proposed as a therapeutic option for human patients.

Sox9 is known to be a marker of reactive BECs, and we recently demonstrated that enhanced Sox9b expression inhibited LPC-to-hepatocyte differentiation [34]. As *sox9b* is a downstream target gene of Notch signaling in the liver [25], we demonstrate here that reduction of *sox9b* expression promotes LPC-to-hepatocyte differentiation. Although Sox9 is known to regulate biliary development [24, 25] and ectopic expression of Sox9 is observed in hepatocytes during several forms of liver injury [26–30], less is known about the role of Sox9 in liver regeneration. Expression of Sox9 in hepatocytes is thought to promote a bipotent state [28, 30]. This is also the case in the context of liver cancer; Sox9 expression in hepatocellular carcinoma (HCC) cells was associated with a more undifferentiated state, venous invasion, and reduced overall survival in patients [47]. Additionally, nuclear localization of Sox9 in intrahepatic cholangiocarcinoma (ICC) correlated with moderately or poorly differentiated status [48], and Sox9 overexpression in ICC was associated with increased invasiveness and poorer prognosis [49]. Notch may also drive the expression of Sox9 in ICC, as hydrodynamic tail vein injection of a NICD plasmid with an AKT overexpression plasmid was sufficient to induce ICC in mice, and the resulting ICC expressed Sox9 [50]. As patients with chronic liver disease are more likely to develop liver cancer [1], reduction of Sox9 expression as a means to promote LPC-to-hepatocyte differentiation in patients with chronic liver disease may also reduce liver tumorigenesis. However, the importance of Sox9 in promoting hepatocyte proliferation in models of chronic liver injury is unknown; thus, more work is needed before strategies that reduce Sox9 expression can be employed in patients with chronic liver diseases.

In conclusion, our current study demonstrates that inhibition of the Notch-Sox9 signaling axis promotes LPC-to-hepatocyte differentiation in zebrafish. Our findings support the importance of this signaling pathway in LPC-mediated liver regeneration and prove the utility of the zebrafish liver injury model to identify the modulators of LPC proliferation and differentiation. We anticipate that future studies will continue to elucidate the factors that regulate LPC differentiation during regeneration and thereby will lead to the development of new therapeutic strategies for patients with chronic liver diseases.

## 4. Materials and Methods

### 4.1. Zebrafish Studies

Experiments were performed with the approval of the Institutional Animal Care and Use Committee (IACUC) at the University of Pittsburgh. Embryos and adult fish were raised and maintained under standard laboratory conditions. We used the *sox9b^fh313^* mutant line and following transgenic lines: *Tg(fabp10a:CFP-nfsB)^s931^* [4], *Tg(EPV.TP1-Mmu.Hbb:hist2h2l-mCherry)^s939^* [35], *Tg(hsp70l:canotch3-EGFP)^co17^* [36], *Tg(EPV.Tp1-Mmu.Hbb:Cre-ERT2,cryaa:mCherry)^s959^* [51], *and Tg(ubb:loxP-eCFP-loxP-sox9b-2A-mCherry)^jh4 37^* (referred to here as *Tg(fabp10a:CFP-NTR)*, *Tg(Tp1:H2B-mCherry)*, *Tg(hs:N3ICD)*, *Tg(Tp1:CreERT2)*, *and Tg(ubb:loxP-CFP-loxP-sox9b-2A-mCherry)*, respectively).

Hepatocyte ablation was performed by treating *Tg(fabp10a:CFP-NTR)* larvae with 10 mM MTZ in egg water supplemented with 0.2% DMSO and 0.2 mM 1-phenyl-2-thiourea (PTU).

### 4.2. Genotyping of *sox9b* Mutants

For *sox9b* genotyping, genomic DNA was amplified with either wild-type allele- (5′-AGACCAGTCGTAGCCCTT-3′) or mutant allele-specific (5′-AGACCAGTCGTAGCCCTA-3′) reverse primer and a common forward primer (5′-TGAGTGTGTCCGGAGCTCCGA-3′).

### 4.3. LY411575, JQ1, DMH1, and MS-275 Treatment

For MS-275 (Selleckchem, Houston, TX) treatment, 25 *μ*M was used for its final concentration. For JQ1 (Cayman Chemical, Ann Arbor, MI), 3 *μ*M was used for final concentration as previously described [52]. For LY411575 (Cayman Chemical, Ann Arbor, MI) and DMH1 (Selleckchem, Houston, TX) treatments, 10 *μ*M was used as previously reported [33].

### 4.4. Whole-Mount Immunostaining

Whole-mount immunostaining was performed as previously described [53], using the following antibodies: goat anti-Hnf4a (1 : 50; Santa Cruz, Dallas, TX), mouse anti-Bhmt (1 : 400; gift from Jinrong Peng at Zhejiang University), rat anti-mCherry (1 : 400; Allele Biotechnology, San Diego, CA), and Alexa Fluor 488-, 568-, and 647-conjugated secondary antibodies (1 : 500; Life Technologies, Grand Island, NY).

### 4.5. Image Acquisition, Processing, and Statistical Analysis

A Zeiss LSM700 confocal microscope was used to obtain image data. Confocal stacks were analyzed using the Zen 2009 software. All figures, labels, arrows, scale bars, and outlines were assembled or drawn using the Adobe Illustrator software. For analyses concerning only two groups, a two-tailed Student's *t*-test was performed, with *P* < 0.05 considered significant. For analyses concerning more than two groups, a one-way ANOVA test was performed, with *P* < 0.05 considered significant. Quantitative data were shown as means ± SEM.

### 4.6. Heat-Shock Condition

*Tg(hs:N3ICD)* larvae were heat-shocked by transferring them into egg water prewarmed to 37°C and kept at this temperature for 20 minutes as previously described [54].

### 4.7. Cre/loxP-Mediated Sox9b Overexpression

Fish carrying the *Tp1:CreERT2* transgene were crossed to *Tg(ubb:loxP-CFP-loxP-sox9b-2A-mCherry)* fish. Larvae from the crosses were treated with MTZ from 3.5 to 5 dpf and additionally treated with both 5 *μ*M 4-OHT from 2 to 5 dpf for 3 days. At 6 dpf, 24 hours after 4-OHT and MTZ washout, the larvae were harvested and processed for immunostaining to reveal mCherry^+^ cells that express Sox9b, as previously described [37].

### 4.8. Quantitative Polymerase Chain Reaction (qPCR)

Total RNA was extracted from 50 dissected livers (pooled) using the RNeasy Mini Kit (Qiagen, Valencia, CA); cDNA was synthesized from the RNA using the SuperScript® III First-Strand Synthesis SuperMix (Life Technologies, Grand Island, NY) according to the kit protocols. qPCR was performed as previously described [55], using the Bio-Rad iQ5 qPCR machine with the iQ™ SYBR Green Supermix (Bio-Rad, Hercules, CA). *eef1a1l1* was used for normalization as previously described [32]. Technical replication (3X) was performed with cDNA samples. The primers used for qPCR are as follows:

*eef1a1l1* forward (5′-CTGGAGGCCAGCTCAAACAT-3′), *eef1a1l1* reverse (5′-ATCAAGAAGAGTAGTACCGCTAGCATTAC-3′), *fabp10a* forward (5′-GCAGGTTTACGCTCAGGAGA-3′), *fabp10a* reverse (5′-TCCTGATCATGGTGGTTCCT-3′); *bhmt* forward (5′-CTGATCGCTGAGTACTTTG-3′), *bhmt* reverse (5′-CAATGAAGCCCTGGCAGC-3′), *sox9b* forward (5′-CTGATCGCTGAGTACTTTG-3′); *sox9b* reverse (5′-CACACCGGCAGATCTGTTT-3′), *tfa* forward (5′-ACTACGCTGTGGCTGTTGTG-3′); *tfa* reverse (5′-AATCCTTTGCCCAGTCCTTT-3′), *her9* forward (5′-AATGCCAGCGAGCATAGAAAGTC-3′), and *her9* reverse (5′-TGCCCAAGGCTCTCGTTGATTC-3′).

## Data Availability

The data used to support the findings of this study are available from the corresponding author upon request.

## Abbreviations

LPC: Liver progenitor cell
BEC: Biliary epithelial cell
Sox9: SRY-related HMG box transcription factor 9
N3ICD: Notch3 intracellular domain
Rbpj: Recombination signal-binding protein immunoglobulin kappa J
DDC: 3,5-diethoxycarbonyl-1,4-dihydrocollidine
BET: Bromodomain and extraterminal
BMP: Bone morphogenetic protein
Hdac1: Histone deacetylase 1
NTR: Nitroreductase
MTZ: Metronidazole
dpf: Days postfertilization
h: Hours
4-OHT: 4-Hydroxytamoxifen
HCC: Hepatocellular carcinoma
ICC: Intrahepatic cholangiocarcinoma
IACUC: Institutional Animal Care and Use Committee
DMSO: Dimethyl sulfoxide
PTU: 1-Phenyl-2-thiourea
qPCR: Quantitative polymerase chain reaction.
